# Electromagnetic Interference Shielding of Metal Coated Ultrathin Nonwoven Fabrics and Their Factorial Design

**DOI:** 10.3390/polym13040484

**Published:** 2021-02-03

**Authors:** Sundaramoorthy Palanisamy, Veronika Tunakova, Shi Hu, Tao Yang, Dana Kremenakova, Mohanapriya Venkataraman, Michal Petru, Jiri Militky

**Affiliations:** 1 Department of Material Engineering, Faculty of Textile, Technical University of Liberec, 46117 Liberec, Czech Republic; veronika.tunakova@tul.cz (V.T.); shi.hu@tul.cz (S.H.); dana.kremenakova@tul.cz (D.K.); mohanapriya.venkataraman@tul.cz (M.V.); Jiri.Militky@tul.cz (J.M.); 2 Institute for Nanomaterials, Advanced Technologies and Innovation, Technical University of Liberec, 46117 Liberec, Czech Republic; tao.yang@tul.cz (T.Y.); michal.petru@tul.cz (M.P.)

**Keywords:** electromagnetic shielding, ultrathin nonwoven, copper-nickel coating, design of experiment, textile material

## Abstract

Electromagnetic (EM) radiation is everywhere in this world and galaxy in different forms and levels. In some cases, human beings need to protect themselves from electromagnetic radiations and the same thing is also recommended for electronic devices as well. Lots of studies are there on the shielding of electromagnetic radiation interference using metals, polymers, and minerals. For protecting the human being, textile structures are playing the main role. In the textile material structure itself many types are there; each one is having its unique geometrical shape and design. In this work, the copper/nickel-coated ultrathin nonwoven fabric is prepared like a strip. The 3, 6, and 9 mm thick strips are prepared and laid at different gaps, angles, and layered to study the effect of factors on EM shielding effectiveness as per ASTM D4935-10 standard. The design of experiment has been done to analyze the three factors and three levels of the strip properties having an influence on electromagnetic shielding results. From the findings of the design of experiment (DoE) screening design, the factors are the thickness of the strips, the gap between the strips, and the strips laid angle having a statistically significant effect on electromagnetic shielding effectiveness.

## 1. Introduction

An electromagnetic (EM) fields are everywhere in this universe; it occurs naturally as well as artificially. Earths’ magnetic fields and lightning are the natural sources of EM field and all electronic devices and electric power transmission lines are the artificial or man-made sources of EM field [1]. EM ionized radiation is divided into four categories, static field, extremely low-frequency EM fields, intermediate frequency EM fields and radio frequency EM fields [2]. The intermediated and radio frequency EM radiations maybe harmful to living beings as well as electronic devices and also it travels at the speed of light. Living beings exposed to an extreme level of EM radiations may cause cancer, tissue damage, lymphoma, leukemia etc., [3] and the electronic devices are damaged with EM radiations because it creating electromagnetic interference (EMI) [4]. The shielding of the living beings and the electronic devices are important to protect from EM radiation and its interferences.

Metals are the best material for EM shielding applications which are silver, copper, stainless steel, iron, gold, nickel, brass, graphite, etc., are good conductors of electricity as well as reflect and absorb most of the EM radiations [5]. It is having some drawbacks like it is heavier, able to get corrode, not flexible etc. The use of metal-coated metal textile materials is overcoming the drawbacks of the metal properties i.e., it becomes flexible, less weight, and porous. Particularly in textile materials different techniques are used to convert the conventional textile material to conductive textile material; the techniques used are coating conductive polymers [6,7], metal coating of fiber, yarn & fabric [8,9], metal fibers blending [10,11], metal core wires [12,13], and carbonization of fabric, yarn & fibers [14].

Textile material itself has many different structures and designs, the major structures are woven, knitted, and nonwoven. Many studies are there on textile structures and designs on EM shielding. Kan Lai et al. [15] used the four metals (silver (Ag), copper (Cu), titanium (Ti), and aluminum(Al) to coat on the surface of the polyester (PET) filament using the vacuum evaporation deposition technique and produced woven fabric and tested it for electromagnetic shielding effectiveness (EM SE) as per ASTM D 4935 method. At 2.45 GHz frequency the Ag/PET, Cu/PET, Al/PET, and Ti/PET is shown as 80 dB, 72 dB, 64 dB, and 26 dB SE respectively for approximately 30 µm thickness of the metal coating. The Ag and Cu has very good EM SE among the set of metals. Jung-Sim Roh et al. [16] used the PET/Cu/PET filament covered yarn and PET/stainless steel (SS)/PET filament covered yarn to produce the woven plain fabric at different opening. The Cu yarn fabric has SE of 46 dB at 1.5 GHz frequency and the SS yarn fabric has 41 dB at 1.5 GHz frequency. For SS yarn fabric, without the grid, grid opening area of 0.16, 0.63 and 2.53 mm^2^ has SE of 41, 36, 32 and 26 dB at 1.5 GHz respectively. This study concluded that, the EM SE value can change by using the grid structure for composite applications. Kai Yang et al. [17] have developed the Cu/Ni coated Milife nonwoven fabric using the electroless plating method for EM shielding application. At maximum 20 wt% Ni/80 wt% Cu coated fabric has a total SE of 25 dB at 1.5 GHz frequency among it 14 dB was reflected radiation (SE_R_).

Some of the works in textile materials studied the increase in the number of layers and its angle against the EM SE and it shows interesting results. Zhi-Cai Yu et al. [18] studied the warp knitted fabric along with conductive weft yarn was inserted to produce the fabric using the crochet machine. The warp yarn is polyester and the weft yarns are the rubber yarn, crisscross section PET (CSP), antibacterial nylon (AN) wrap yarns, stainless steel wire (SSW) as core (CSP/AN/SSW) hybrid yarn A and polyester yarn, bamboo charcoal polyester yarn (BC-PET), cotton yarn and jade fiber yarn as hybrid yarn B. EM SE of the warp knitted fabric prepared with CSP/AN/SSW and BP-PET as weft yarn and PET as warp yarn has 10 dB at 1.5 GHz frequency. The fabric was laid at two layers and three layers and the laid angles are 0°/0°, 0°/0°/0°, 0°/90°, 0°/90°/0°, 0°/45°, and 0°/45°/0°. For two-layer and three-layer fabric the laid angle 0°/90° and 0°/90°/0° has highest SE value of 28 and 40 dB at 3 GHz frequency. Laying angle of 90° has more efficient for EM shielding. Olena Kyzymchuk et al. [19] prepared the SS wire and cotton yarn 1 × 1 rib knitted fabric using a flat knitting machine. Single-layer and double-layer fabric samples are tested for EM SE and the result for 0°/0° and 0°/90° has higher SE than the single layer that is 3–5 dB and 10–15 dB at 1.5 GHz frequency respectively.

In this work, the copper-coated Milife^®^ fabric named as the ultrathin nonwoven fabric was taken and cut into strips to study the effect of the gap, thickness and angle of the strips on the EM SE. The EM SE is measured as per the ASTM standard [20]. To compare the effect of the parameters on EM SE, the design of experiment (DoE) more precisely screening design is implemented. MiniTab^®^ software is used for the analysis of DoE.

### Electromagnetic Shielding Mechanism

An EM field is the combination of electric *E* and magnetic *H* fields that are perpendicular to each other. Due to voltage differences, the electric field is creating and its moving charges are creating the magnetic field. The current contains the electric as well as magnetic fields. There are two regions in the electromagnetic interference (EMI) shielding; the near field shielding region and the far-field shielding region. If the distance from the source is less than the wavelength (*λ*) divided by *2π* is called a near or induction field and greater distance is called a far or radiation field. The reduction in the electric and magnetic field is caused by shielding because the EM wave is reflected from the shield surface, multiple reflections in-between the shield and absorption of the shield. The EMI shielding is mainly based on far-field radiation, so the plane wave is used for measurement. In this case, the multiple reflection loss is neglected because the reflecting surface is larger than the skin depth, *δ* (m), is defined as (Equation (1))

(1) $$\delta =\frac{1}{\sqrt{\pi fµK}}$$

where, *f* (Hz) is frequency and *µ* is the magnetic permeability equal to *µ*_0_. *µ_r_*, *µ*_0_ is the absolute permeability of free space (vacuum, *µ*_0_ = 4π 10^−7^ H/m) and *K* (S m^−1^) is the electrical conductivity. An electric field at a high frequency penetrates only the near-surface region of a conductor. The amplitude of the wave decreases exponentially as the wave penetrates the conductor. The depth at which the amplitude is decreased to *1/e* of the value at the surface is called the “skin depth,” and the phenomenon is known as the “skin effect” [4].

EMI shielding value is represented in decibel (dB) and its effectiveness is mentioned by total shielding effectiveness *SE_T_*, in (Equation (2)):

(2) $$SE_{T}=\;-10log\;\left[\frac{P_{2}}{P_{1}}\right]$$

where, *P*_1_ is power without shield specimen (W/m^2^), *P*_2_ is power with the presence of shield specimen (W/m^2^) and log x is decimal logarithm [21].

EM SE of the specimen is influenced by its electrical conductivity, permeability, and permittivity, properties of ambient surroundings, and parameters of the EM source. There are many studies in textile materials that related the EM SE with electrical conductivity [22,23,24,25], and opening area [15,26,27] of the specimen. The EM SE is measured by various methods and its varying according to the specimen application. These EM SE testing techniques are open field test [28], coaxial transmission line test [20,29,30], shielded box test [31] and shielded room test [32].

The main motivation of this work is to simulate the conductive material structural parameters against the electromagnetic shielding property. EM shielding is needed in many industrial as well as commercial applications and each one needs a various level of shielding effectiveness (ref. Tables 4 and 5). According to the requirement, the use of conductive materials can be optimized using the proposed model and thereby make the design of the shielding counter more effective. “Effectively” in this means of cost wise as well as resources wise. In this experiment, the copper/ nickel-coated ultra-thin nonwoven material strips were used for modeling. The simulated structures were formed by the conductive strips’ material at the different thickness, gap, and angle to study their effects on electromagnetic shielding effectiveness. Another goal is to find out the optimal model based on the requirement.

## 2. Materials

The copper/nickel (Cu/Ni) coated ultrathin nonwoven fabric (Cu/Ni NW) (Milife^®^) named as ‘MEFTEX 20’ was procured from the BOCHEMIE a.s., Bohumin, Czechia. Milife^®^ is the registered trademark of JX NIPPON ANCI Corporation, Chiba, Japan, contains 100% polyester nonwoven made with the combined orientation of spinning technology. The Milife^®^ fabric is thin, smoothy and it has a silk-like appearance achieved from laying perpendicular polyester filaments. Metallization of Cu/Ni on ultrathin nonwoven (Milife^®^) was done with chemical and electrochemical metal deposition method using ‘roll on roll’ coating technique. The fabric is also finished with anti-corrosion resistance on the metal surface. The ‘Cu/Ni NW’ fabric parameters are given in Table 1.

**Figure 1:**
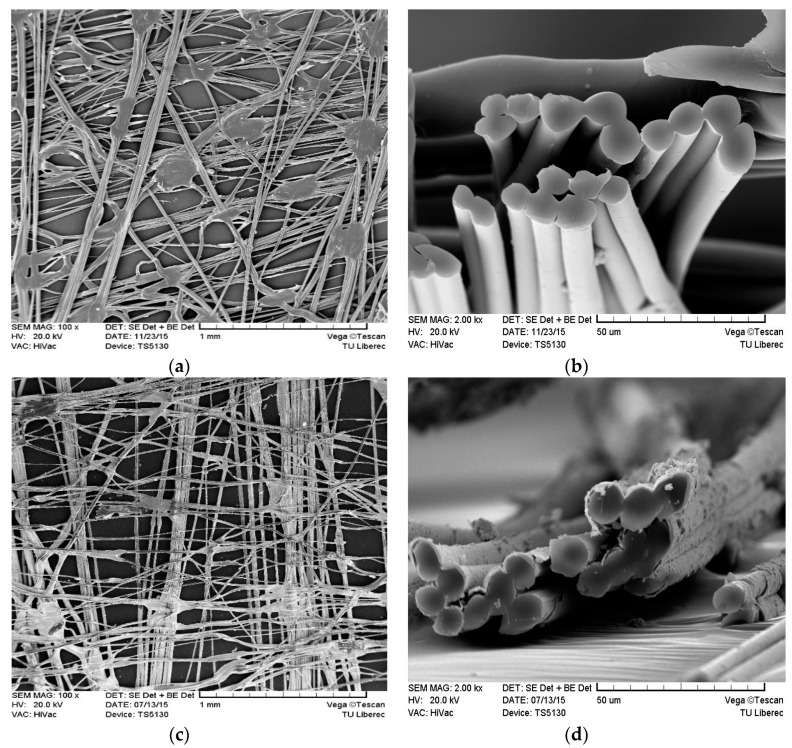
SEM image of the ultrathin nonwoven fabric (**a**) at 100× magnification, (**b**) cross-section view at 2000× magnification, SEM image of Cu/Ni coated ultrathin nonwoven fabric (**c**) at 100× magnification, (**d**) cross-section view at 2000× magnification.

The scanning electron microscopic images of the uncoated and coated ultrathin nonwoven fabric are shown in Figure 1. Coating of the material is evenly applied on the nonwoven surface see Figure 1c,d.

**Figure 2:**
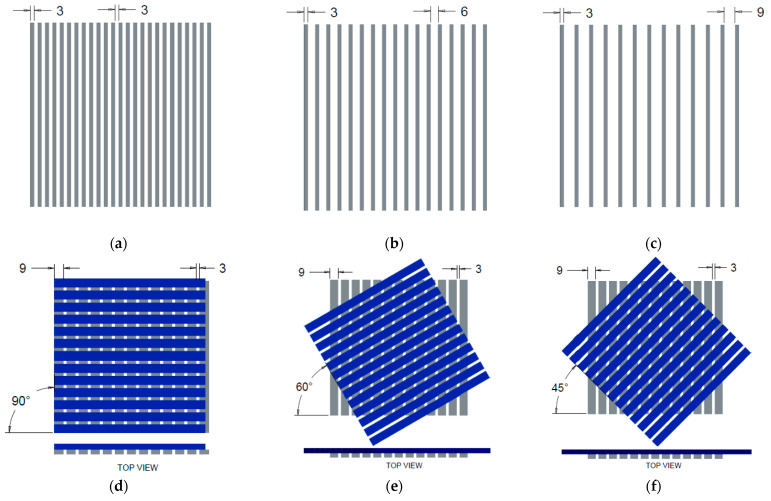
The graphical images of the single-layer Cu/Ni NW strips of 3 mm thickness at distance (**a**) 3 mm (SS30) (**b**) 6 mm (SS60) and (**c**) 9 mm (SS90) and two-layer Cu/Ni NW strips of 9mm thickness laid at (**d**) 90° (TL390) (**e**) 60° (TL360) and (**f**) 45° (TL345) angles.

Many studies are about the effect of EM SE on the gap between the conductive material in textile structures but there are very few studies for the gap between and angle of laying conductive material in textile structures [18,19]. In this study, the ‘Cu/Ni NW’ sample is cut into strips at 3, 6 & 9 mm thick and laid with 3, 6, & 9 mm gap between each other. Each thickness of strips was laid at the above gaps and form a single layer of the sheet as shown in Figure 2 and with help of CREO^®^ CAD, the graphical model of strips was created. For example, the single-layer 3 mm thick strip was laid at 3, 6, & 9 mm gap was shown in Figure 2a–c. Likewise, 3 mm thick strips, 6 mm and 9 mm thick strips were laid at 3, 6, & 9 mm gap. Two-layer strips were formed by laying two single layer strips at 90°, 60° & 45° angle with same gap, 9 mm thick strips of 3 mm gap laid at 90°, 60° & 45° angle are shown in Figure 2d–f. Moreover, 9 mm thick strips of 6 mm and 9 mm gap are laid at 90°, 60° & 45° angle to form two-layer strip samples. Likewise, 9 mm thick strips, the 3 mm and 6 mm thick strips were formed as two-layer strip samples with various gap and laid angle. The two-layer samples were taken for the EM SE test as per ASTM method to analyze the effect of gap between and angle between the conductive strips’ material. Each thickness of the strips laid in 9 different ways and tested for EM SE. So, a total of 27 samples were prepared from the combination of strip thickness, gap between the strips, and angle between the two layers of strips for testing EMI shielding and the sample codes or names are given in Table 2. The overlaying of the strips at various angles is prepared with the same strip thickness as well as the same gap between the strips as shown in Figure 2d–f. For reference, the one-layer and two-layer strips laid at 0° angle was prepared for EM SE test and the sample code is given in Table 2.

## 3. Methods

### 3.1. Experimental Design

To examine the main factors and their interactions, the factorial design was chosen. Factors are varied together in the experimental strategy, instead of one at a time [34]. Design of experiment (DoE) methods include the factorial experiments, overcome the limitations of the trial-and-error method described above, and quickly give the kind of understanding and results that are needed. The primary goal is usually to show the statistical significance of an effect that a particular factor exerts on the dependent variable and to find the optimum setting of factors to get the desired level, small variability of a dependent variable, or to minimize uncontrollable variables.

Screening factorial design (SFD) was chosen to completely and systematically study the interaction between factors in addition to identifying significant factors. In this experiment, there are three variables and three levels used to design an experimental study. The factors and levels of the factorial design are listed in Table 3. The factorial experimental design was analyzed using Minitab^®^ software.

The variable levels are mentioned in ‘−1’ as lower, ‘0’ as a medium, and ‘+1’ as a high value for response. The strip thickness and gap between the strips level are 3, 6, and 9 mm mentioned as lower, medium and higher. For laid angles of strips at 45°, 60°, and 90° is mentioned as lower, medium, and higher levels.

### 3.2. Electromagnetic Shielding Effectiveness

SE of the sample set was measured according to the ASTM D4935-10 [20], for the planar materials using a plane-wave, the far-field EM wave at the temperature T = 21 °C, and the relative humidity RH = 54%. SE of the samples was measured over the frequency range of 30 MHz to 1.5 GHz. The set-up consisted of a sample holder with its input and output connected to the network analyzer. A shielding effectiveness test fixture (model EM-2107A, Electro-Metrics Corporation, Johnstown, NY, USA) was used to hold the sample. The design and dimension of the sample holder follow the ASTM method mentioned above. A vector analyzer Rohde & Schwarz ZN3 was used to generate and receive the electromagnetic signals. The standard mentioned above determines the shielding effectiveness of the fabric using the insertion-loss method. A reference measurement for the empty cell was required for the shielding effectiveness assessment. A “through” calibration with the help of the reference sample was made first. A load measurement was performed on a solid disk shape sample subsequently. The reference and load specimens must be of the same material and thickness. Both the reference and load samples geometries are according to the ASTM D 4935-10. The measurements were performed at five different places of the strip samples because of the subsequent statistical analysis.

According to the requirements of EM shielding textiles [35], depending on professional or general use, textiles can be classified into five grades from a fair grade to an excellent grade (see Table 4 and Table 5). Professional use comprises professional protective uniforms for electronic manufacturers, shielding of medical equipment, etc., whereas general use is represented by casual wear, maternity clothes, aprons, shielding of consumptive electronic products and communication- related products, etc.

## 4. Results and Discussion

### 4.1. Electromagnetic Shielding Effectiveness of Cu/Ni NW Samples

**Figure 3:**
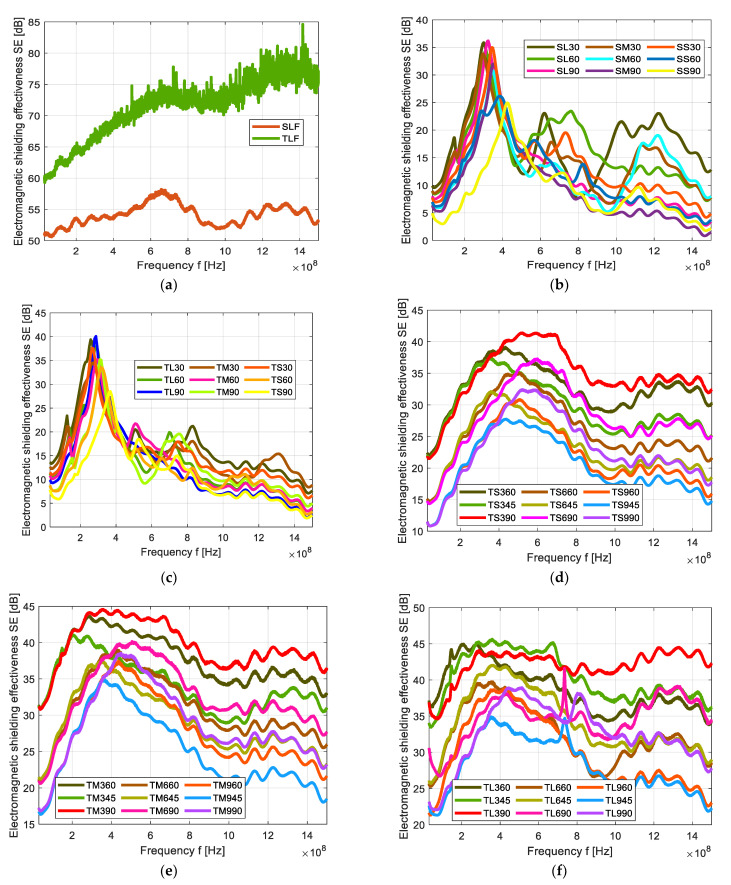
Electromagnetic shielding effectiveness (EM SE) versus frequency of the Cu/Ni NW sample (**a**) single layer (SLF) & two-layer (TLF), (**b**) single layer strips, (**c**) two layers strips laid at 0° angle, (**d**) two layers of 3 mm thick strips laid at 45°, 60° and 90° angles, (**e**) two layers of 6 mm thick strips laid at 45°, 60° and 90° angles, and (**f**) two layers of 9 mm thick strips laid at 45°, 60° and 90° angles.

EM SE of Cu/Ni NW fabric and its strips samples are tested with coaxial transmission line method as per ASTM standard [20] and the results are shown in Figure 3. All the samples were tested from 30 MHz to 1.5 GHz frequency and the SE is shown in dB.

Figure 3a shows the frequency-dependent EM SE of single layer fabric (SLF) and two-layer fabric (TLF) samples of Cu/Ni NW. SLF fabric is a precursor used for the preparation of strips for further sample design. The two-layer sample has the highest SE value of 74 dB at 1.5 GHz frequency and the SE is increasing logarithmically with increasing frequency. For the SLF sample, the SE is about 52 dB at 30 MHz as well as at 1.5 GHz frequency, this result is showing a “very good” SE (see Table 4) for all measured frequencies.

Figure 3b shows the frequency-dependent EM SE of single layer strips (SL, SM & SS) samples of Cu/Ni NW. For the lowest frequency (30 MHz), SE for all samples is between 5 and 10 dB. For the highest frequency SE for all samples is between 2 and 13 dB. It is possible to observe a global maximum of SE around 350–400 MHz frequency, where SE rises to 25–35 dB. It seems that with increasing strip thickness, the frequency global maximum is shifting to a higher frequency. Similarly, with increasing distance between the strips, the frequency of global maximum is shifting to a lower frequency. When checking 1.5 GHz frequency, the highest ability to shield electromagnetic field (about 13 dB) has sample SL30 (sample having the thickest strips and the smallest distance between them). Sample SM90 (sample with a medium thickness of strips and the highest distance between them) has the lowest SE (<3 dB) at the same frequency. SE is decreasing with the increasing gap between the strips as shown in Figure 3b e.g., for samples SL90, SL60, and SL30 made of 9 mm thick strips having 3 mm, 6 mm and 9 mm gaps between the strips have SE value of 3 dB, 7.7 dB and 12.7 dB at 1.5 GHz frequency respectively.

Figure 3c shows the frequency-dependent EM SE of two-layer strips (TL, TM & TS) at laid at 0° angle samples of Cu/Ni NW. For the lowest frequency (30 MHz), SE for all samples is between 6 and 14 dB. For the highest frequency SE for all samples is between 2 and 8 dB. It is possible to a observe global maximum of SE around 250–400 MHz frequency, where SE rises to 28–40 dB. It seems that with increasing strip thickness, the frequency global maximum is shifting to a higher frequency. Similarly, with increasing distance between the strips, the frequency of global maximum is shifting to a lower frequency. When checking 1.5 GHz frequency, the highest ability to shield electromagnetic field (about 8 dB) has sample TM30 (sample having the medium-thick strips and the smallest distance between them). Sample TL90 (sample with the highest thickness of strips and the highest distance between them) has the lowest SE (<3 dB) at the same frequency. SE is decreasing with the increasing gap between the strips as shown in Figure 3c e.g., for samples TL90, TL60, and TL30 made of 9 mm thick strips having 3, 6 and 9 mm gaps between the strips have SE value of 6.6, 3 and 2.5 dB at 1.5 GHz frequency respectively.

Cu/Ni NW samples made of 3 mm thick strips were prepared of two-layers (TS) using various gaps between strips as well as various laying angle and their SE [dB] versus frequency graph is shown in Figure 3d. From this graph, the SE is increasing with increasing angles and a decrease in the gap. All the two-layer strip samples show that the SE is increasing steeply to the frequency range of 400–600 MHz, where the global maximum of SE (from 27 to 42 dB for all TS samples) is observable. This frequency range corresponds to wavelength 50–75 cm. After that, a slow decrease follows. For the lower frequencies, the SE is between 10 and 23 dB, whereas the lowest SE samples TS945, TS960, and TS990. The highest SE at frequency 30 MHz is reported for samples TS345, TS360, and TS390 which have a 3 mm distance between the strips. It is visible that for low frequencies (<400 MHz) the effect of layering angle is negligible for all studied samples. When checking the highest frequency, SE is in the range 15–33 dB and the highest SE is achieved by sample TS390 having the lowest distance between conductive strips and the layering angle 90°. It seems that layering at 90° is preferable, securing the highest SE in each sample group.

Figure 3e shows SE versus frequency of the two-layers (TM) Cu/Ni NW strip samples. In this case, the strip thickness is 6mm. Observing the SE frequency dependence, SE is steeply increasing from the starting frequency till the 200–400 MHz where the global maximum of SE for all samples is located (SE = 35–45 dB). After the initial increase, SE is decreasing slowly with a further increase in frequency. It is visible, that for the lowest frequency, the layering angle has almost no effect. At 30 MHz frequency, groups of samples with the same strip thickness and the same distance between strips have the same SE, namely 17, 21, and 31 dB for samples with 3, 6, 9 mm gaps between the strips respectively. The effect of layering angle is manifested when the frequency around 200 MHz is exceeded. When studying the highest frequency (1.5 GHz), SE is in the range from 17 to 36 dB, whereas the lowest SE shows a sample with the highest gaps between strips (9 mm) and 45° layering angle and the highest SE shows a sample with the lowest gaps between strips (3 mm) and the 90° layering angle. It was confirmed that layering at 90° is preferable also in this sample group.

In Figure 3f shows the SE versus frequency graph for 9 mm thick strips of Cu/Ni NW sample of two-layers (TL). Observing the SE frequency dependence, SE is steeply increasing from the starting frequency till the 200–450 MHz where the global maximum of SE for all samples is located (SE = 37–46 dB). Another peak of SE is formed between 980 MHz to 1.25 GHz frequency and it has SE of 18–35 dB range. When studying the highest frequency (1.5 GHz), SE is in the range from 22 to 42 dB, whereas the lowest SE shows a sample with the highest gaps between strips (9 mm) and 45° layering angle and the highest SE shows a sample with the lowest gaps between strips (3 mm) and the 90° layering angle.

TS and TM samples are acting well against the medium-range frequencies but the TL samples are “very good” against lower range and “good” against higher range frequencies. At 90° the contact angle of strip layers has a very good SE compare with 45° and 60°. When compared with 3- and 6-mm thickness of the strips, 9 mm thickness has very good SE at higher frequencies.

### 4.2. Electromagnetic Shielding Effectiveness of Cu/Ni NW Strip Samples at 1.5 GHz Frequency

**Figure 4:**
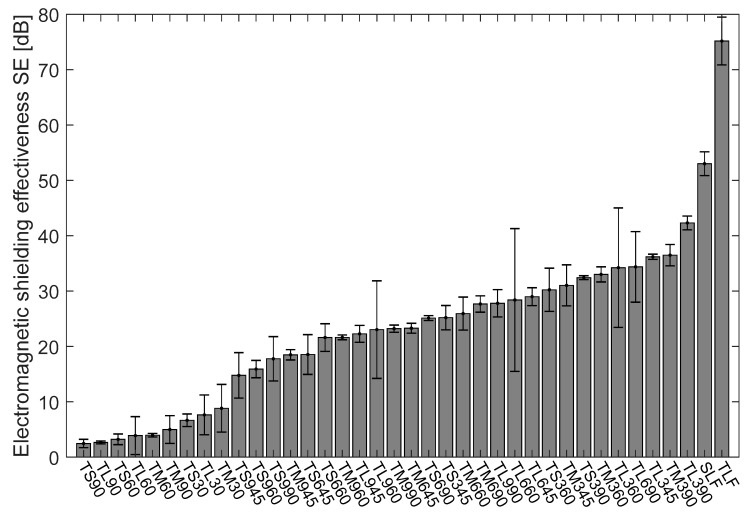
Comparison of the mean value of the electromagnetic shielding effectiveness at 1.5 GHz frequency for Cu/Ni NW strip samples with 95% confidence interval of mean.

There is a broad frequency range in EM but the 1.5 GHz frequency is the widely used frequency in most communication devices. Figure 4 shows the ascending order arrangement of the Cu/Ni NW strip samples’ electromagnetic shielding effectiveness in dB at 1.5 GHz frequency. According to the requirement of EM shielding textile [35], depending on professional or general use, textiles can be graded with five categories from poor to excellent (see Table 4 and Table 5).

The mean value of SE of the Cu/Ni NW fabrics and its strip samples were calculated with 95% confidence interval (CI) of mean and plotted in a bar chart (Figure 4). In the bar graph, the SE values are arranged in ascending order to understand easily. Among the set of strip samples, the samples laid at a 90° angle is showing the highest SE at 1.5 GHz frequency compare with 0°, 45° and 60°. All the two-layer strips laid at 0° angle is showing less than 10 dB SE at 1.5 GHz frequency. TL390 has the highest SE among all the samples and it has a ‘Good’ grade for professional use and an “Excellent” grade for general use (See Table 4 and Table 5). Most of the angular laid TL samples are having SE values above 27 dB and TL960 and TL945 have 22 and 23 dB at 1.5 GHz frequency. Variation in SE value is only because of the area covered by the strip, for angular laid TL samples, the cover geometry of the gap is different in the open area. At 90° angle, the diagonal length of the gap between the strips is lower than that of 60° and 45° angles are in Figure 2. This case is applicable for angular laid TS and TM samples too. TS945 sample has the lowest value of SE (14.77 dB) at 1.5 GHz frequency rated as a “Good” grade for general use. Of course, the SE is directly proportional to the area covered by the sample so the TS9 series samples have very less cover area compare with TS6 and TS3 series samples. TL345 and TM390 samples have the SE value of 36.19 and 36.48 dB showing no significant difference between them but the CI at 95% was 6.36 and 0.43 respectively because of the higher aperture area for TL345. The SLF and TLF samples have SE of 53 and 75 dB at 1.5 GHz frequency respectively.

**Figure 5:**
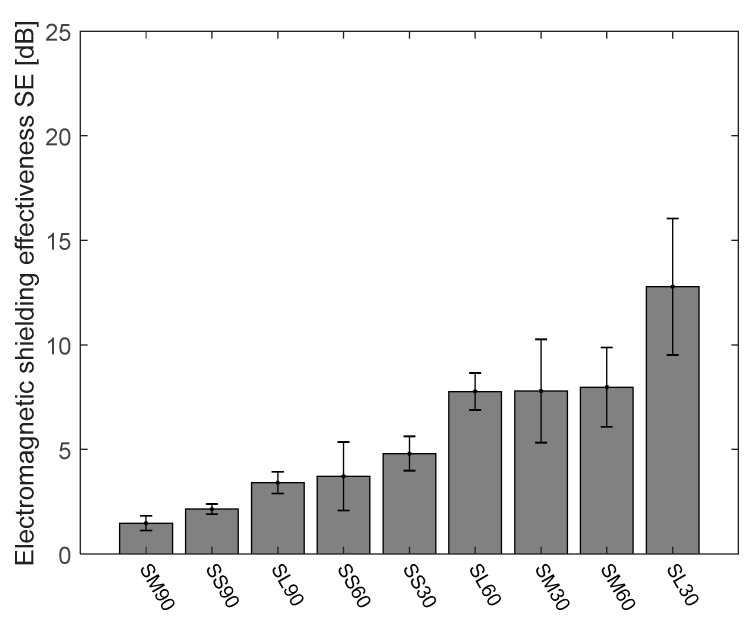
Bar chart of EM SE at 1.5 GHz of the single-layer strip samples with 95% confidence interval of mean.

The EM SE at 1.5 GHz of single layer strip samples with a different gap (3, 6 & 9 mm) and different thicknesses (SS, SM & SL) is shown in Figure 5. From this bar chart, the EM SE of the samples is arranged in ascending order, the SL30 sample has the highest SE of 12.78 dB. The lowest SE of 1.47 dB is recorded for the SM90 sample. A huge difference in the SE value is mainly because of the area covered by the strips. Further, the detailed analysis of the effect of the area covered on EM SE is analyzed.

### 4.3. Effect of Electromagnetic Shielding Effectiveness on the Cover Area of Strips

**Figure 6:**
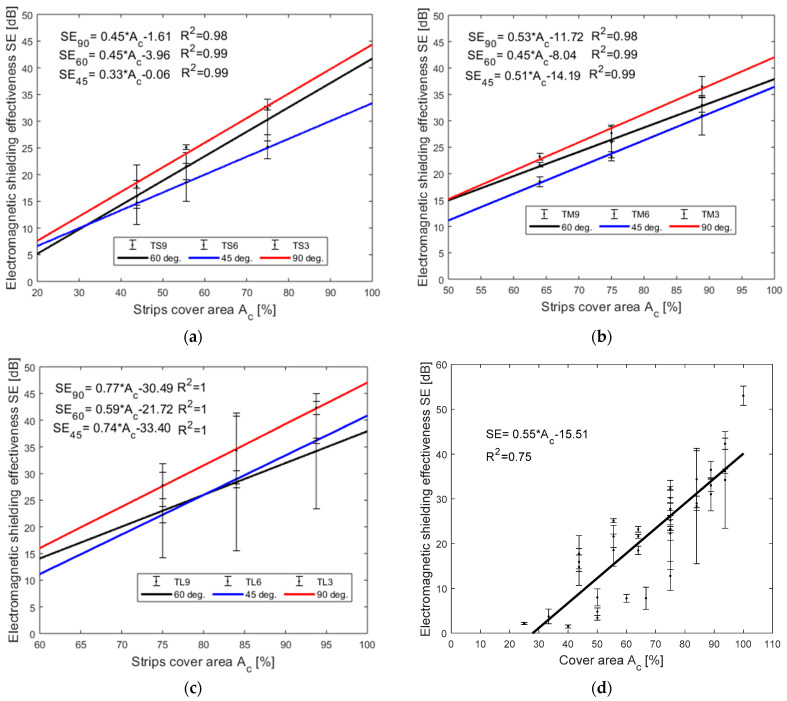
Statistical analysis of EM SE (dB) at 1.5 GHz frequency versus cover area (A_c_) (%) of (**a**) TS samples (**b**) TM samples (**c**) TL samples and (**d**) one layer, two-layer strip samples and SLF sample.

The cover area of the two layers strip samples was calculated using models (see Figure 2) created with help of CREO^®^ CAD software. Figure 6 is showing the EM SE (dB) versus the percentage area covered by the strips (A_c_) (%) for all the two layers of strip samples. Figure 6a–c is showing the coefficient of determination (R^2^) above 0.99, which means the predictability of SE value is highly accurate with the cover area (A_c_). The SE versus A_c_ of the TS samples is shown in Figure 6a in which the strips laid at an angle of 90° has higher SE value than 60° and 45°. Variation in SE for the same cover area is occurred because of the higher area per aperture for 60° and 45° laying angle of strips compared with 90° angle. In the same case of TS samples, SE value variation is seen for the TM and TL samples (see Figure 6b,c) but the SE of TL series has a higher value compared with the TM series and TS series for the same angles. At all angles, the TL series samples have higher *A_c_* compare with TS and TM series samples; TL390 samples have 93% cover area compare with TS390 and TM390 has 75% and 88% cover area. Moreover, the cover area increases by the decrease in the gap between the strips; TL990 has a 75% cover area compare with TS390 having 93% cover area. Figure 6d is showing the combined graph of EM SE versus percent cover area of TS, TM, TL, series and SLT samples. SLT sample is having a 100% cover area and it has the highest SE of 53 dB. In general, the SE is increasing with an increase in the cover area but for the same cover area, there can be different SE values. This is because of the aperture area variation. In this case, the higher aperture area has slightly less SE than the lower aperture area, it can be seen at all the readings in Figure 6. From the linear regression analysis shown in Figure 6d SE increases with increasing cover area and the coefficient of determination (R^2^) is 0.75. The prediction of SE with respect to the cover area for this model is average. Hence, the increases in the strips laid angle with increases in SE value as seen clearly in Figure 6a–c.

### 4.4. Influence of Area per Aperture at Different Laying Angle and its Effect on SE Results

**Figure 7:**
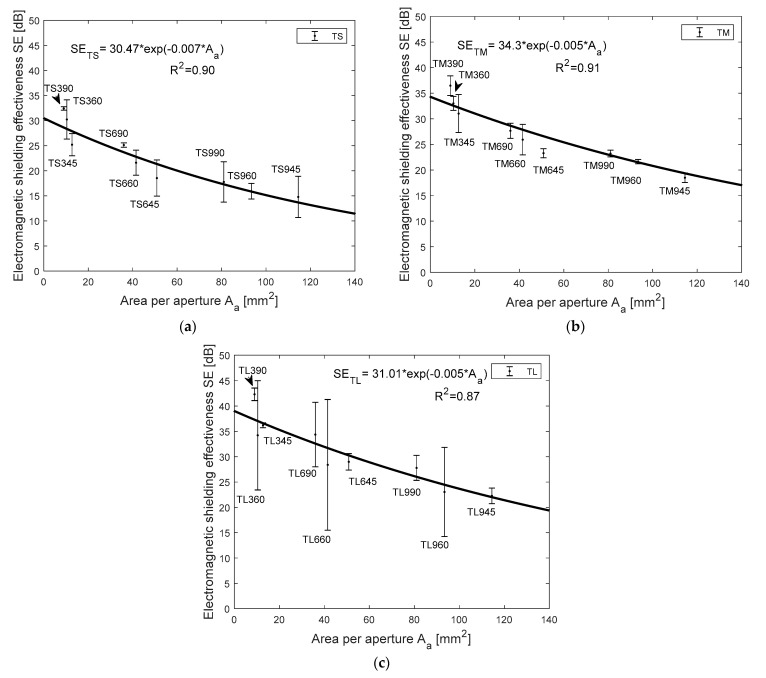
Graph of shielding effectiveness (SE) (dB) at 1.5 GHz frequency versus area per aperture (A_a_) (mm^2^), two-layer strips of (**a**) 3 mm thickness samples, (**b**) 6 mm thickness samples, and (**c**) 9 mm thickness samples.

Beyond the cover area of the strip samples, another factor that influences the SE value is the area per aperture (A_a_) or open space in the samples. The cover factor graph (Figure 6) shows that the same percent cover area has different SE values. After analyzing the images (Figure 2), the number of aperture and area of aperture is varying for each angle of layering. So, the EM SE at 1.5 GHz frequency versus area per aperture was plotted and statistically analyzed and it is shown in Figure 7. SE versus A_a_ of TS samples is shown in Figure 7a. SE is increasing with a decrease in area per aperture and the regression model has coefficient of determination (R^2^) of 0.91 for the exponential trend line. Regression analysis is proving that SE is also influenced by area per aperture. Figure 7b,c for TM and TL samples also are showing the same trend line for SE versus Aa like TS samples and the regression model has a coefficient of determination (R^2^) of 0.91 and 0.87 respectively. Irrespective of thicknesses of the two layers strip samples, the laid angle is varying the area per aperture; at 90° angular laid strips have very less A_a_ compare with 45° and 60° laid angles (see Figure 7). The maximum A_a_ is achieved at a 45° laid angle of strips and it registered lower SE compare with other laid angles. Other results noted in this study show that the increases in the gap between strips also decreases the SE value. The total cover area or total aperture area was calculated and compared against the EM SE in Figure 6 graphs and the area per single aperture was calculated and compared against the EM SE in Figure 7 graphs. The change in EM SE of same cover area samples is mainly because of various area per single aperture values i.e., wider opening in the samples causes the reduction in EM SE value. For example, TL945, TL960, and TL990 samples cover area is the same (75%), and the area per aperture is 114 mm^2^, 93 mm^2^ and 81 mm^2^ respectively but the EM SE values are 22.2, 23, and 27.8 dB respectively. Hence, the change in EM SE value for the same cover area of samples is because of various area per aperture and was proved that EM SE decreases with area per aperture increase.

### 4.5. Design of Experiment

DoE model consists of the three factors at three levels as shown in Table 3 and the SFD model is taken for the analysis. In the SFD model, there are 13 base runs and 39 total runs in the design.

#### 4.5.1. Analysis of Variance

Analysis of variance (ANOVA) of the SFD model is shown in Table 6. ANOVA test is performed for identifying the factors which have a significant effect on SE at 1.5 GHz frequency. The ratio of the mean square and its error was examined by the ANOVA test. *F*-value is the variance between the means of two populations and *p*-value is the probability associated with the *F*-value. In Table 6, the three main factors, its square, and its cross effects are analyzed and calculated for *F*-value and *p*-value. If the *p*-value is less than the significant level (α = 0.05) then the factor is statistically significant on SE value and vice versa. ANOVA test shows the factors lesser than significant level are A, B, and C and all other factors are higher than the significant level.

Equation (3) is showing the regression equation in terms of uncoded units of the SFD model, SE value is predicted with help of this regression equation and R^2^ for prediction is 92.23% (Table 6) which is proving that the predictability of SE is highly possible.

#### 4.5.2. Regression Equation in Uncoded Units

SE = 25.932 + 4.446 A − 7.113 B + 2.729 C − 0.209 A × A+ 1.240 B × B + 0.463 C × C − 0.334 A × B+ 0.267 A × C − 0.517 B × C(3)

#### 4.5.3. Standardized Effect Charts

**Figure 8:**
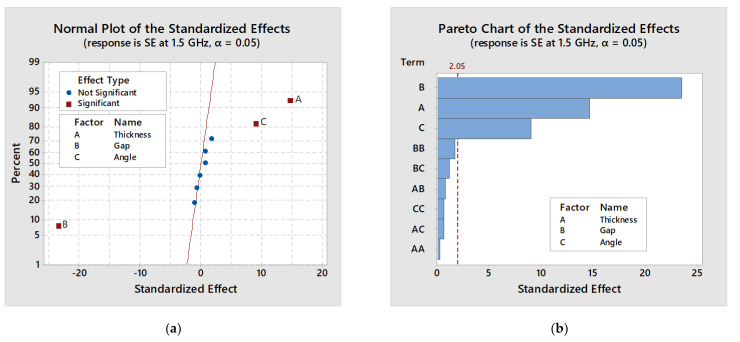
The normal plot of the (**a**) standardized effects and (**b**) standardized Pareto chart.

The standardized effects and Pareto chart of the factors and their combinations are represented in Figure 8 at a 5% significance level (α = 0.05) for SE at 1.5 GHz frequency. In the Pareto chart (Figure 8b), standardized effect values of the significant factors and their interactions are higher than the critical value. Although; the Pareto chart is helpful to compare the absolute value of the effects of each factor and its interactions of the considered SFD, a normal plot of the standardized effect is used to determine the significance and insignificant of each effect of factors more accurately. The factors with a significant difference in SE value is plotted in red marks and factors without significant difference is plotted in blue marks as seen in the graph (Figure 8a). In a normal probability plot of effect (Figure 8a), the factors A, and C have a positive significant effect on SE, and factor B has a negative significant effect on SE. Among positive effects the thickness of the strips (A) has the most significant difference on SE value, i.e., SE is increasing with increasing thickness of the strip. The negative effect is saying that the SE is increasing with a decrease in the gap between the strips (B). The factors AA, BB, BC, AC, CC and AB are showing no significant difference in SE value which are all very close to the straight line i.e., normal distribution.

#### 4.5.4. Main Effect Plot

**Figure 9:**
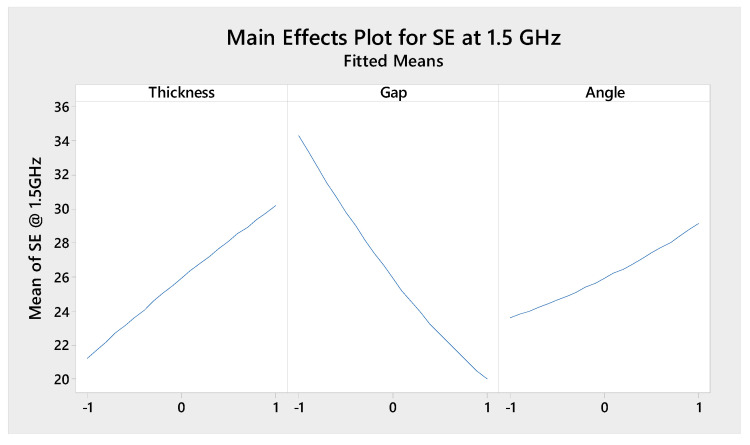
Main effects plot of the factors.

Figure 9 is showing the main effects plot for SE (dB) at 1.5 GHz frequency for the factors A, B, and C. The plot is mainly showing the effects of the factors and their levels on SE value. Factors, A (thickness) and C (angle) is showing a positive effect on SE value in the main effect plot, factor B (gap) has a negative effect on SE value. An increase in thickness and angle is increasing the SE value and an increase in gap is decreasing in SE value. The factor B has more influence on SE value, next influential factor is A and least influential factor is C.

#### 4.5.5. Interaction Plot

**Figure 10:**
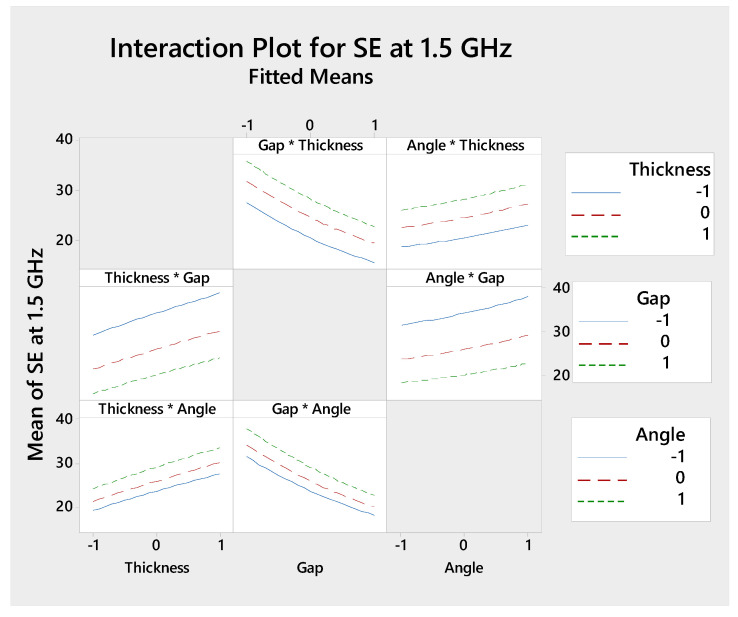
Interaction plot for SE (dB) at 1.5 GHz for the combination of two factors.

Figure 10 is showing the interaction plot for SE (dB) at 1.5 GHz frequency to visualize the factors and their interactions. The interaction plot is mainly indicating the presence of interaction between the factors; the parallel line indicates there is no interaction, out of parallel line indicates interactions, and the cross line indicates more interaction. Moreover, this plot helps to achieve the highest SE value from the best interaction combination of the factors. The highest SE is achieved with a higher level of angle (i.e., 90°) and a lower level of gap (i.e., 3 mm) as shown in the gap*angle interaction plot (Figure 9). SE versus frequency (Figure 3d) of samples TL390 also indicates the same interaction. For interactive effects of gap*thickness and gap*angle, SE decreases with an increasing gap. For thickness*gap and thickness*angle, SE increases with increasing thickness. The angle*thickness and angle*gap interaction show the increase of SE with an increase in angle (from 45° to 90°). However, the interaction plot is reflecting the main plot effects but the results are precisely for each factors interaction.

#### 4.5.6. Contour Plot

**Figure 11:**
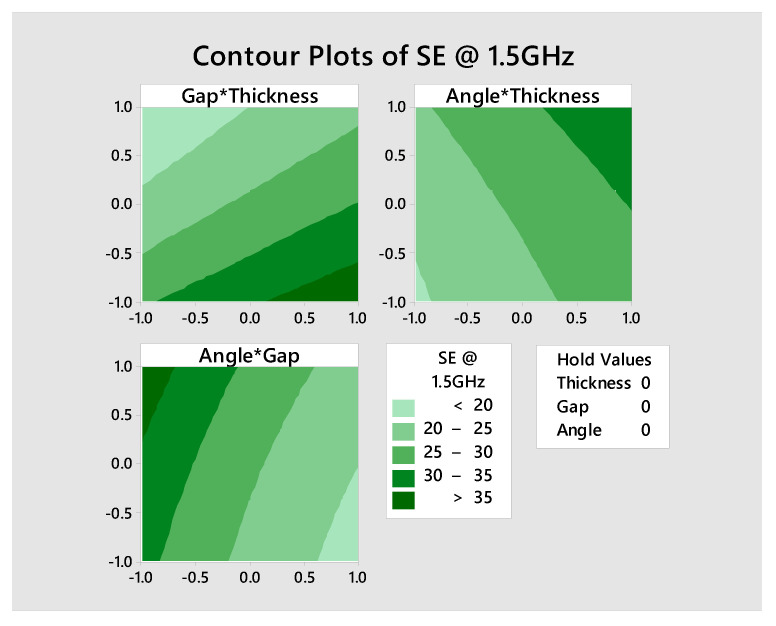
Contour plot showing the quantitative effect of gap and thickness; angle and thickness; angle and gap on SE (dB) at 1.5 GHz frequency.

In Figure 11, the contour plots of SE at 1.5 GHz frequency of the interactive effect of factors are given. Response surface plot (Figure 11) shows how response relates to two continuous design factors, while holding multiple factors in the model at a specified level. The SE > 35 dB is achieved by the lower gap (3 to 4.2 mm) and higher thickness of strip (6 to 9 mm) by holding angle at the medium level (60°). The SE of 30 dB to 35 dB is achieved by the higher angle (60° to 90°) and higher thickness (6.8 to 9 mm) by holding the gap at a medium level (6 mm). Above 35 dB of SE is obtained by the higher angle (72° to 90°) and lower gap (3 to 3.7 mm) by holding thickness at a medium level (6 mm).

## 5. Conclusions

The Copper/Nickel coated 100% polyester ultrathin nonwoven fabric was taken in this study. For reference purposes, the single-layer and two-layer fabric samples were tested for EM SE and it exhibits 53 and 73 dB at 1.5 GHz frequency and graded as ‘very good’ according to the general requirement [35]. All the single layer strips samples (SL) and two-layer strips (TL) laid at 0° angle samples had the EM SE of less than 12.5 and 9 dB respectively at 1.5 GHz frequency. Among TL samples, TL390 exhibits the highest SE of 42 dB at 1.5 GHz frequency. The EM SE (at 1.5 GHz frequency) is increasing with an increase in percent strips cover area (A_c_) for the TL samples; A linear correlation between A_c_ and EM SE has been found (0.98 ≤ R^2^ ≤ 1). A maximum A_c_ of 93.75% for TL3 series samples and recorded the highest SE value. An increase in area per aperture (A_a_) of the TL samples has decreased in EM SE value; An exponential relationship of A_a_ and EM SE was found (0.87 ≤ R^2^ ≤ 0.91). The TL945, TL960, and TL990 samples have A_c_ of 75% but A_a_ of 114, 93, and 81 mm^2^ which has EM SE of 22, 23, and 28 dB respectively at 1.5 GHz frequency. So, the decrease in SE for the same A_c_ of samples is because of the decrease in A_a_. The influence of percent cover area and area per aperture parameters has a significant effect on EM SE results.

The screening factorial design (SFD) model from the design of experiment (DoE) technique is used for the analysis of factors having a significant effect on EM SE at 1.5 GHz frequency. The three main factors are thickness (A), gap (B), and laid angle (C) of the strips at three levels were used for DoE analysis. The SFD model has 13 base runs and 39 total runs in the experiment. In ANOVA, the significant effect of SE on factors was calculated with reference to P-value. Good predictability of EM SE with the regression equation model of factors was found (R^2^ = 0.92). The factors which are A, B, and C has a significant effect on the SE value has found in the Pareto chart; In a normal probability plot, it found that the factors A, and C has positive significant effect and factor B has negative significant effect on SE value. The main effect plot fits confirms that the SE value was increased with an increase in factor A, decreases with increases in factor B, and decreases initially then increases with increases in factor C. The best SE value with respect to factors interaction was found in the interaction plot, the highest SE of 38 dB was found in the interaction of higher C (i.e., 90°) and a lower B (i.e., 3 mm).

Hence, the higher percent cover area and lower area per aperture structure have been recommended for achieving higher EM SE. DoE is concluded that the combination of the larger strip thickness, the lower gap between the strips, and the higher laid angle of strips has an excellent EM SE. This model could be helpful to construct the optimal fabric or composite structures based on the required level of shielding for electromagnetic shielding application.

## Figures and Tables

**Table 1:** Cu/Ni NW fabric parameters and their values.

Parameters *	Values
Areal density (g/m^2^)	24.56 ± 2.69
Thickness (mm)	0.087 ± 0.006
Tensile strength in MD (N/50 mm)	25 [33]
Tensile strength in CD (N/50 mm)	16 [33]
Surface resistivity (Ω)	2.1 ± 0.28

* Areal density, thickness and surface resistivity are measured as per the ASTM method. Note: MD means ‘machine direction’ and CD means ‘cross direction’.

**Table 2:** Cu/Ni NW sample and its code.

No. of Layers	Angle of Layers (°)	Strip Thickness (mm)			Gap between the Strips (mm)
		3	6	9	
One	0	SS30	SM30	SL30	3
		SS60	SM60	SL60	6
		SS90	SM90	SL90	9
Two	0	TS30	TM30	TL30	3
		TS60	TM60	TL60	6
		TS90	TM90	TL90	9
	45	TS345	TM345	TL345	3
	60	TS360	TM360	TL360	
	90	TS390	TM390	TL390	
	45	TS645	TM645	TL645	6
	60	TS660	TM660	TL660	
	90	TS690	TM690	TL690	
	45	TS945	TM945	TL945	9
	60	TS960	TM960	TL960	
	90	TS990	TM990	TL990	
Single-layer of fabric		SLF			
Two-layers of fabric		TLF			

**Table 3:** Factors and its levels used for SFD.

Factors	Levels			Response
Strip thickness (A)	−1	0	+1	EMI SE (dB)
Gap between the strips (B)	−1	0	+1	
Angles of layers (C)	−1	0	+1	

**Table 4:** Classification of EM SE values based on professional use.

Grade	Excellent	Very Good	Good	Moderate	Fair
Range (dB)	SE > 60	60 ≥ SE > 50	50 ≥ SE > 40	40 ≥ SE > 30	30 ≥ SE > 20

**Table 5:** Classification of EM SE values based on general use.

Grade	Excellent	Very Good	Good	Moderate	Fair
Range (dB)	SE > 30	30 ≥ SE > 20	20 ≥ SE > 10	10 ≥ SE > 7	7 ≥ SE > 5

**Table 6:** Analysis of Variance and regression coefficient.

Source	DF	Adj SS	Adj MS	*F*-Value	*p*-Value
Model	9	2355.05	261.67	94.92	0.000
Linear	3	2334.18	778.06	282.24	0.000
Thickness	1	592.96	592.96	215.09	0.000
Gap	1	1517.86	1517.86	550.59	0.000
Angle	1	223.36	223.36	81.02	0.000
Square	3	9.33	3.11	1.13	0.354
Thickness*Thickness	1	0.22	0.22	0.08	0.779
Gap*Gap	1	7.81	7.81	2.83	0.103
Angle*Angle	1	1.09	1.09	0.40	0.534
2-Way Interactions	3	8.96	2.99	1.08	0.371
Thickness*Gap	1	1.59	1.59	0.58	0.453
Thickness*Angle	1	1.02	1.02	0.37	0.549
Gap*Angle	1	3.82	3.82	1.39	0.249
Error	29	79.95	2.76	-	-
Lack-of-Fit	3	12.99	4.33	1.68	0.196
Pure Error	26	66.96	2.58	-	-
Total	38	2435.00		-	-
S	R-sq	R-sq(adj)	R-sq(pred)	-	-
1.66035	96.72%	95.70%	94.25%	-	-

* DF—Degree of freedom, SS—Sum of the square, MS—Mean square.

## Data Availability

Not applicable.

## References

[1] Barnes F.S., Greenebaum B. (2006). Bioengineering and Biophysical Aspects of Electromagnetic Fields.

[2] Martin R. (2008). Epidemiology of Electromagnetic Fields.

[3] Lin J.C. (2012). Electromagnetic Fields in Biological Systems.

[4] Ott H.W. (2009). Electromagnetic Compatibility Engineering.

[5] Geetha S., Kumar K.K.S., Rao C.R.K., Vijayan M., Trivedi D.C. (2009). EMI shielding: Methods and materials—A review. J. Appl. Polym. Sci..

[6] Kaynak A., Håkansson E. (2009). Characterization of conducting polymer coated fabrics at microwave frequencies. Int. J. Cloth. Sci. Technol..

[7] Yanılmaz M., Saraç A.S. (2014). A review: Effect of conductive polymers on the conductivities of electrospun mats. Text. Res. J..

[8] Uzun S., Han M., Strobel C.J., Hantanasirisakul K., Goad A., Dion G., Gogotsi Y. (2021). Highly conductive and scalable Ti3C2T -coated fabrics for efficient electromagnetic interference shielding. Carbon.

[9] Wu X., Chen Y., Liang K., Yu X., Zhuang Q., Yang Q., Liu S., Liao S., Li N., Zhang H. (2020). Fe_2_O_3_ Nanowire Arrays on Ni-Coated Yarns as excellent electrodes for High Performance Wearable Yarn-Supercapacitor. J. Alloys Compd..

[10] Šafářová V., Militký J. (2014). Electromagnetic shielding properties of woven fabrics made from high-performance fibers. Text. Res. J..

[11] Liang R., Cheng W., Xiao H., Shi M., Tang Z., Wang N. (2018). A calculating method for the electromagnetic shielding effectiveness of metal fiber blended fabric. Text. Res. J..

[12] Ortlek H.G., Saracoglu O.G., Saritas O., Bilgin S. (2012). Electromagnetic shielding characteristics of woven fabrics made of hybrid yarns containing metal wire. Fibers Polym..

[13] Perumalraj R., Nalankilli G., Balasaravanan T.R., Roshanraja K., Shyamsundar G., Dasaradan B.S. (2010). Electromagnetic shielding tester for conductive textile materials. Indian J. Fibre Text. Res..

[14] Li T.-T., Wang R., Lou C.-W., Lin M.-C., Lin J.-H. (2013). Manufacture and effectiveness evaluations of high-modulus electromagnetic interference shielding/puncture resisting composites. Text. Res. J..

[15] Lai K., Sun R.-J., Chen M.-Y., Wu H., Zha A.-X. (2007). Electromagnetic Shielding Effectiveness of Fabrics with Metallized Polyester Filaments. Text. Res. J..

[16] Roh J.-S., Chi Y.-S., Kang T.J., Nam S.-W. (2008). Electromagnetic Shielding Effectiveness of Multifunctional Metal Composite Fabrics. Text. Res. J..

[17] Yang K., Periyasamy A.P., Venkataraman M., Militký J., Kremenakova D., Vecernik J., Pulíček R. (2020). Resistance against Penetration of Electromagnetic Radiation for Ultra-light Cu/Ni-Coated Polyester Fibrous Materials. Polymers.

[18] Yu Z.-C., He H., Lu Y., Zhang J.-F., Lou C.-W., Chen A.-P., Lin J.-H. (2015). Functional Properties and Electromagnetic Shielding Behaviour of Elastic Warp-knitted Fabrics. Fibres Text. East. Eur..

[19] Tunakova V., Tunak M., Bajzik V., Ocheretna L., Arabuli S., Кизимчук О.П., Vlasenko V. (2020). Hybrid knitted fabric for electromagnetic radiation shielding. J. Eng. Fibers Fabr..

[20] (2010). D4935-10 ASTM Standard Test Method for Measuring the Electromagnetic Shielding Effectiveness of Planar Materials.

[21] Vojtech L., Neruda M. (2013). Design of radiofrequency protective clothing containing silver nanoparticles. Fibres Text. East. Eur..

[22] Palanisamy S., Tunakova V., Militky J. (2017). Fiber-based structures for electromagnetic shielding – comparison of different materials and textile structures. Text. Res. J..

[23] Kardarian K., Busani T., Osório I., Domingos H., Igreja R., Franco R., Cortez J. (2014). Sintering of nanoscale silver coated textiles, a new approach to attain conductive fabrics for electromagnetic shielding. Mater. Chem. Phys..

[24] Engin F.Z., Usta I. (2013). Electromagnetic shielding effectiveness of polyester fabrics with polyaniline deposition. Text. Res. J..

[25] Tian M., Du M., Qu L., Chen S., Zhu S., Han G. (2017). Electromagnetic interference shielding cotton fabrics with high electrical conductivity and electrical heating behavior via layer-by-layer self-assembly route. RSC Adv..

[26] Safarova V., Militký J. (2013). Electromagnetic Field Shielding Fabrics with Increased Comfort Properties. Adv. Mater. Res..

[27] Šafářová V., Militký J. (2015). Multifunctional metal composite textile shields against electromagnetic radiation-effect of various parameters on electromagnetic shielding effectiveness. Polym. Compos..

[28] Romero S.F., Rodríguez P.L., Bocanegra D.E., Martinez D.P., Cancela M.A. (2018). Comparing Open Area Test Site and Resonant Chamber for Unmanned Aerial Vehicle’s High-Intensity Radiated Field Testing. IEEE Trans. Electromagn. Compat..

[29] Valente R.V., De Ruijter C., Vlasveld D., Van Der Zwaag S., Groen W. (2017). Setup for EMI Shielding Effectiveness Tests of Electrically Conductive Polymer Composites at Frequencies up to 3.0 GHz. IEEE Access.

[30] Ozen M.S., Sancak E., Beyit A., Usta I., Akalin M. (2012). Investigation of electromagnetic shielding properties of needle-punched nonwoven fabrics with stainless steel and polyester fiber. Text. Res. J..

[31] Krzysztofik W.J., Borowiec R., Bieda B. (2011). Some consideration on shielding effectiveness testing by means of the nested reverberation chambers. Radioengineering.

[32] Duran D., Kadoğlu H. (2014). Electromagnetic shielding characterization of conductive woven fabrics produced with silver-containing yarns. Text. Res. J..

[33] MEFTEX 20. https://www.meftex.cz/en/meftex-20/p-3/.

[34] Kenett R.S., Zacks S., Amberti D. (2014). Modern Industrial Statistics.

[35] (2005). FTTS-FA-003 Specified Requirements of Electromagnetic Shielding Textiles.

